# Regenerating Skeletal Muscle Compensates for the Impaired Macrophage Functions Leading to Normal Muscle Repair in Retinol Saturase Null Mice

**DOI:** 10.3390/cells11081333

**Published:** 2022-04-13

**Authors:** Nastaran Tarban, Hajnalka Halász, Péter Gogolák, Éva Garabuczi, Alexander R. Moise, Krzysztof Palczewski, Zsolt Sarang, Zsuzsa Szondy

**Affiliations:** 1 Doctoral School of Molecular Cell and Immune Biology, Faculty of Medicine, University of Debrecen, 4032 Debrecen, Hungary; nastarantarban88@gmail.com (N.T.); halasz.hajnalka@med.unideb.hu (H.H.); 2 Department of Immunology, Faculty of Medicine, University of Debrecen, 4032 Debrecen, Hungary; gogy@med.unideb.hu; 3 Department of Biochemistry and Molecular Biology, Faculty of Medicine, University of Debrecen, 4032 Debrecen, Hungary; gevi@med.unideb.hu (É.G.); sarang@med.unideb.hu (Z.S.); 4 Medical Sciences Division, Northern Ontario School of Medicine, Sudbury, ON P3E 2C6, Canada; amoise@nosm.ca; 5 Department of Ophthalmology, Gavin Herbert Eye Institute, University of California, Irvine, CA 92697, USA; kpalczew@uci.edu; 6 Department of Basic Medical Sciences, Faculty of Dentistry, University of Debrecen, 4032 Debrecen, Hungary

**Keywords:** cardiotoxin injury, retinol saturase, neuropeptide Y, MFG-E8, efferocytosis, skeletal muscle repair

## Abstract

Skeletal muscle repair is initiated by local inflammation and involves the engulfment of dead cells (efferocytosis) by infiltrating macrophages at the injury site. Macrophages orchestrate the whole repair program, and efferocytosis is a key event not only for cell clearance but also for triggering the timed polarization of the inflammatory phenotype of macrophages into the healing one. While pro-inflammatory cytokines produced by the inflammatory macrophages induce satellite cell proliferation and differentiation into myoblasts, healing macrophages initiate the resolution of inflammation, angiogenesis, and extracellular matrix formation and drive myoblast fusion and myotube growth. Therefore, improper efferocytosis results in impaired muscle repair. Retinol saturase (RetSat) initiates the formation of various dihydroretinoids, a group of vitamin A derivatives that regulate transcription by activating retinoid receptors. Previous studies from our laboratory have shown that RetSat-null macrophages produce less milk fat globule-epidermal growth factor-factor-8 (MFG-E8), lack neuropeptide Y expression, and are characterized by impaired efferocytosis. Here, we investigated skeletal muscle repair in the tibialis anterior muscle of RetSat-null mice following cardiotoxin injury. Our data presented here demonstrate that, unexpectedly, several cell types participating in skeletal muscle regeneration compensate for the impaired macrophage functions, resulting in normal muscle repair in the RetSat-null mice.

## 1. Introduction

Skeletal muscle is frequently injured, but temporary damage is compensated by the remarkable regenerative capacity of this tissue [1]. The regeneration process involves several interrelated phases. In the first inflammation phase, tissue-resident macrophages (Mϕs) sensing the damage initiate a local, sterile inflammation and recruit further immune cells to the injury site. Then, quiescent myogenic stem cells, called satellite cells (SCs), become activated and differentiate into myoblasts, which proliferate and fuse. Finally, in the tissue remodeling phase, the growth of muscle fibers, resolution of inflammation, revascularization, and innervation of new fibers take place [2].

In the inflammation phase, the first wave of arriving immune cells, neutrophils secrete chemokines and pro-inflammatory cytokines to recruit blood-derived monocytes. Following tissue entry, these monocytes differentiate into Ly6C^+^ M1 polarized pro-inflammatory Mϕs, which produce further inflammatory mediators, such as interleukin (IL)-6, IL-1, tumor necrosis factor- (TNF)-α, and nitric oxide (NO), which activate quiescent SCs to proliferate and differentiate into myoblasts [3,4]. Mфs also clear the necrotic myofibers and dying neutrophils in the injured tissue. This efferocytosis process polarizes them to Ly6C^−^ M2-like healing Mϕs, which guide the resolution of inflammation and full tissue regeneration [5]. To do so, healing Mϕs secrete anti-inflammatory lipid mediators [6] and cytokines, such as IL-10, thereby limiting inflammation and favoring myoblast differentiation [7]. These cells also release growth factors, e.g., transforming growth factor (TGF)-β, which induces new extracellular matrix formation [8], and the TGF-β family member growth differentiation factor (GDF)3, which specifically stimulates myogenic cell fusion to form new myofibers [9]. Thus, Mϕs play a central role in guiding the whole muscle-regeneration process, and the timed switch between their two main subsets, the inflammatory and healing Mϕs, is key to proper repair [10]. Increasing evidence indicates that when efferocytosis and consequently the Mϕ polarization fail, regeneration also fails. Thus, loss of efferocytosis receptors, such as scavenger receptor class BI [11], Mer tyrosine kinase [12], and integrin β3 coreceptor transglutaminase (TG)2 [13], or loss of the macrophage polarization mediating transcription factor peroxisome proliferator-activated receptor (PPAR)γ [9] have been reported to result in impaired skeletal muscle repair following cardiotoxin (CTX)-induced injury.

Interestingly, both efferocytosis [14] and myoblast fusion [15] require the appearance of phosphatidylserine (PS) on the cell surface and involve the same PS binding receptors. Thus, the efferocytosis receptors brain angiogenesis inhibitor (BAI)1 [16], stabilin-2 [17], TG2 [13], Axl [12], and integrin β3 [18] were all found to participate in myoblast differentiation and fusion. Some of the PS receptors directly bind PS, while others use bridging molecules to form a link with PS. Integrins belong to the second group by using milk fat globule-epidermal growth factor-factor 8 (MFG-E8) as a bridging molecule [19]. Both Mϕs [19] and myoblasts [20] secrete MFG-E8, which binds with its RGD domain to the integrin receptor, and with its gamma carboxylated glutamate side chains to PS [21].

Retinol saturase (RetSat) is an oxidoreductase enzyme that catalyzes the conversion of all-trans retinol to all-*trans*-13,14-dihydroretinol [22]. All-*trans*-13,14-dihydroretinol is oxidized in vivo to all-*trans*-13,14-dihydroretinoic acid, a selective agonist of the retinoic acid receptor, and possibly to 9-*cis*-13,14-dihydroretinoic acid [23], which is identified as a physiologically relevant retinoid X receptor agonist [24]. Recent work in our laboratory has shown that the loss of RetSat alters Mϕ differentiation at the monocyte stage, resulting in Mϕs which express less MFG-E8 and practically no neuropeptide Y (NPY) [25]. As a consequence of less MFG-E8 production, RetSat-deficient Mϕs show impaired phagocytic capacity [25]. Due to the impaired macrophage efferocytosis, and also due to the fact that both MFG-E8 and NPY would have anti-inflammatory activity by promoting Mϕ polarization [19,26,27], loss of RetSat results in the development of systemic lupus erythematosus-like autoimmunity in aged female mice, similar to other mice that show impaired efferocytosis [28]. In the present study, we investigated whether the loss of RetSat affects the ability of skeletal muscle to be repaired in mice.

## 2. Materials and Methods

### 2.1. Reagents

All reagents were obtained from Sigma-Aldrich (Budapest, Hungary), except where indicated otherwise.

### 2.2. Experimental Animals

Experiments were carried out using 2–6-month-old young adult male C57BL/6J RetSat^+/+^ mice and their full-body RetSat^−/−^ littermates [29]. Mice were bred in heterozygous form under specific pathogen-free conditions in the central animal facility of the University of Debrecen. To check whether the RetSat^−/−^ mice are indeed full knock out, we determined the NEO cassette expression by qRT-PCR in several tissues and found it to be expressed (Supplementary Figure S1). All animal experiments were approved by the Animal Care and Use Committee of the University of Debrecen, with permission numbers 7/2016 and 7/2021/DEMÁB.

### 2.3. Cardiotoxin-Induced Muscle Injury Model

Mice were anesthetized with 2.5% isoflurane using a SomnoSuite device. The muscle damage was induced by injecting into the tibialis anterior (TA) muscle 50 μL of 12 μM CTX (Latoxan, Valence, France), dissolved in phosphate-buffered saline (PBS). This concentration of CTX induces severe muscle injury, facilitating detection of more significant alterations in the subsequent regeneration process in the absence of regeneration–related genes but still allows full regeneration as detected 3 months after the injury. Mice were sacrificed, and TA muscles were harvested at various time points following injury and processed for further experiments.

### 2.4. Hematoxylin/Eosin and Immunofluorescent Staining of the Regenerating Muscle

TA muscles from control mice or at the indicated days post-injury were dissected for histological assessment. The muscles were snap-frozen in liquid nitrogen-cooled isopentane and kept at −80 °C. Seven micrometer cryosections were cut at −20 °C using a 2800 Frigocut microtome (Leica, St Jouarre, France) and were kept at −20 °C until further analysis. Hematoxylin/eosin (H&E) staining was performed to assess the overall morphology and presence of necrotic fibers following injury. Images from the sections were taken using an AMG EVOS cl microscope (Thermo-Fisher Scientific, Waltham, MA, USA).

To calculate the cross-sectional areas (CSA) and collagen-stained areas, frozen muscle sections were incubated in 10 mM citric acid-sodium citrate buffer (pH 6.0) for 15 min then in blocking solution (50% FBS in PBS) for 1 h at room temperature. After blocking, samples were labeled with Dylight 488 conjugated anti-laminin B (PA5-22901, Invitrogen, Carlsbad, CA, USA) (1:100) and anti-collagen 1 antibody (SAB4500362, Sigma-Aldrich (Budapest, Hungary)) (1:100) at 4 °C overnight followed by Alexa Fluor 488 conjugated Goat anti-Rabbit IgG secondary antibody followed by washing three times with PBS. The nuclei were labeled with 4 μg/mL 4′,6-diamidino-2-phenylindole (DAPI) (Invitrogen, Carlsbad, CA, USA), and the slides were mounted with glass coverslips. Images were taken on a FLoid Cell Imaging Station fluorescent microscope (Thermo-Fisher Scientific, Waltham, MA, USA) and analyzed using ImageJ v1.52 software (National Institutes of Health, Bethesda, MD, USA) with a muscle morphometry plugin. Areas with fibers containing centrally-located nuclei were considered regenerating muscle parts. CSAs are reported in μm^2^, while collagen content is reported as the percentage of the total examined regenerating area.

### 2.5. Quantification of Necrotic Areas

Necrotic myofibers were defined as pink pale patchy fibers infiltrated by basophil single cells and quantified as described previously [11]. Briefly, 4 non-overlapping microscope view field areas were digitally captured from 6–8 H&E stained TA muscle sections at 200-fold magnification. The percentage of necrotic area/relative to the total regenerating area was calculated after manual outlining of the necrotic fibers in the sections.

### 2.6. Isolation of Muscle-Derived CD45^+^ Leukocytes

Muscle-derived CD45^+^ cell isolation was carried out as described previously [12]. Briefly, TA muscles were removed at 2, 3, and 4 days post-injury and dissociated in RPMI containing 0.2% collagenase II (Thermo-Fisher Scientific, Waltham, MA, USA) at 37 °C for 1 h and filtered stepwise through 100 µm and 40 µm filters. CD45^+^ cells were isolated using magnetic sorting (Miltenyi Biotec, Gladbach, Germany).

### 2.7. Generation of Bone Marrow-Derived Macrophages (BMDMs) for NEO Cassette Expression Analysis

Bone marrow progenitors were obtained from the femur of 2 to 4-month-old RetSat ^+/+^ and RetSat^−/−^ mice by lavage with sterile physiological saline. Cells were differentiated for 5 days in DMEM medium supplemented with 10% conditioned medium derived from L929 cells, as a source for macrophage colony-stimulating factor (M-CSF); and 2 mM glutamine, 100 U/mL penicillin, and 100 mg/mL streptomycin at 37 °C in 5% CO_2_. Non-adherent cells were washed away every second day.

### 2.8. Gene Expression Analysis

Total RNA of muscle-derived CD45^+^ cells, BMDMs, and different organs homogenized in TRIzol with a Shakeman homogenizer (BioMedical Science, Tokyo, Japan) were isolated with TRIzol reagent (Invitrogen, Carlsbad, CA, USA), according to the manufacturer’s instructions. Total RNA was reverse transcribed into cDNA using a High Capacity cDNA Reverse Transcription Kit (Thermo-Fisher Scientific, Waltham, MA, USA), according to the manufacturer’s instructions. Quantitative RT-PCR was carried out in triplicate using FAM-labeled MGB assays (Thermo-Fisher Scientific, Waltham, MA, USA) on a Roche LightCycler LC 480 real-time PCR instrument. Relative mRNA levels in the case of CD45^+^ cells were calculated using the comparative C_T_ method and normalized to beta-actin (β-actin) mRNA. In the case of the TA muscle samples, gene expression was normalized to the total RNA content (200 ng) of the samples. Catalogue numbers of the TaqMan assays used were the following: Actb Mm02619580_g1, Tgfb1 Mm01178820_m1, MyoD1 Mm00440387_m1, Myhc1 Mm01332489_m1, Myog Mm00446194_m1, Tnf Mm00443258_m1, Gdf3 Mm00433563_m1, IL1B Mm00434228_m1, IL10 Mm01288386_m1, IL6 Mm00446190_m1, IL4 Mm00445259_m1, PPARg Mm00440940_m1, Arg1 Mm00475988_m1, MFG-E8 Mm00500549_m1, MCP-1 Mm00441242_m1, Pax7 Mm00834082_m1, RetSat Mm00458863_m1, NPY Mm01410146_m1, eNOS Mm01164908_m1, iNOS Mm00440502_m1, NEO cassette NEOCASSETTE.

### 2.9. Quantification of Satellite and Fibrocyte-Adipocyte Progenitor Cells in the TA Muscle Following CTX-Induced Injury

For intramuscular SC and fibro adipogenic precursor (FAP) cell detection, TA was collected at day 4 post-injury and dissociated in RPMI containing 0.2% collagenase II (Gibco/Thermo-Fisher Scientific, Waltham, MA, USA) at 37 °C for 1 hr and filtered through a 100 µm filter. Prior to staining, ~225,000 pcs polystyrene microbeads (8 mm, 78511) were added to the muscle cell suspension samples to determine the absolute cell numbers later. The identification of the muscle precursor cells was based on the α7-integrin (PE-conjugated, 130-120-812, Miltenyi Biotec, Bergisch Gladbach, Germany) staining of the SCs, and the CD140a (BV711 conjugated, 740740, BD Biosciences, San Jose, CA, USA) and Sca1 (BV605 conjugated, 563288, BD Biosciences, San Jose, CA, USA) staining of the FAPs. Other muscle tissue-resident and immigrant cell types were gated from the measurement by specific staining with a moisture of monoclonal antibodies, including biotin anti-mouse CD45 (103104, BioLegend, San Diego, CA, USA), biotin anti-mouse CD31 (102404, BioLegend, San Diego, CA, USA), and biotin anti-mouse Ter119 (79748, BioLegend, San Diego, CA, USA). In the second step, APC-conjugated streptavidin (405207, BioLegend, San Diego, CA, USA) was added to the cells. Before the measurement, cells were washed with 0.5% BSA-physiological saline and suspended in 0.5% BSA-physiological saline supplemented with SYTO16 green-fluorescent nucleic acid stain (S7578, Invitrogen, Carlsbad, CA, USA) (5000× dilution) and 7-AAD non-cell-permeable dead cell stain (A1310, ThermoFisher, Waltham, MA, USA) (1000× dilution) to exclude the injured and dead cells. The measurement was performed with a FACS Aria III cytometer (Becton, Dickinson and Company, Franklin Lakes, NJ, USA) equipped with violet (405 nm), blue (488 nm), yellow (561 nm), and red (633 nm) lasers. The measurement of the microbeads was based on their intense side directional light scattering (SSC) properties. The living cells were gated to CD45/CD31/Ter119 positive and negative populations according to their APC fluorescence. The APC non-stained cells mainly involved the Sca1 bright, CD140a^+^, integrin-α7− FAP cells, and the integrin-α7^+^, Sca1−, CD140a− SC cells. The absolute cell count was based on the ratio of the cells of interest to the microbeads within the measured samples.

### 2.10. Quantification of Intramuscular Immune Cells by Flow Cytometry

The magnetically separated muscle-derived CD45^+^ cells were stained with a combination of Alexa Fluor 488-conjugated anti-F4/80 antibody (MF48020, Invitrogen, Carlsbad, CA, USA) and Alexa Fluor 647-conjugated anti-Ly6G/Ly6C (GR-1) antibodies (108418, BioLegend, San Diego, CA, USA) at room temperature for 15 min. The cells were gated based on their forward and side scatter characteristics. Macrophages were gated as GR-1^–^ and F4/80^+^, while neutrophils as F4/80^–^ and GR-1^+^ cells. This gating strategy was presented in our previous paper [12]. F4/80^+^ macrophages were also analyzed for expression of Ly6C, CD206, or major histocompatibility complex (MHC)II, following staining with the corresponding antibodies, Ly6C PerCP-Cy5.5 (128012, BioLegend, San Diego, CA, USA), CD206-PE (141705, BioLegend, San Diego, CA, USA), or MHCII-FITC (107605, BioLegend, San Diego, CA, USA), respectively. Fluorescent intensity was detected with a Becton Dickinson FACSCalibur instrument (Becton, Dickinson and Company, Franklin Lakes, NJ, USA).

### 2.11. In Vitro Phagocytosis Assay by F4/80^+^ Cells

Phagocytosis experiments were carried out as described previously [30]. Briefly, target C2C12 cells were induced to undergo necrosis by heating the cells at 65 °C for 10 min. Some C2C12 cells were labeled with 1 µM CellTracker Deep Red Dye (ThermoFisher, Waltham, MA, USA), and some were not. Since our previous studies indicated that attenuated efferocytosis of RetSat-null macrophages is related to a decreased production of MFG-E8 during long-term efferocytosis [25], to induce MFG-E8 production, first the non-labeled cells were added to 5-carboxyfluorescein diacetate (CFDA) (6 µM)-stained F4/80^+^ cells isolated from the day 4 CTX-injured collagenase-treated TA muscles by magnetic beads (Miltenyi Biotec, Gladbach, Germany) at 5:1 ratio (dead cell/F4/80^+^ cell) plated in 8-well chamber slides (Gräfelfing, Germany) (3 × 10^5^/well). After 5 h co-culture, F4/80^+^ cells were washed and further incubated with labeled necrotic C2C12 cells for an additional 2 h, after which target cells were washed away extensively. In some cultures, Μϕs were detached by trypsinization, and the percentage of engulfing cells was determined using a Becton Dickinson FACSCalibur flow cytometer (Becton, Dickinson and Company, Franklin Lakes, NJ, USA). Some other cultures were fixed in 1% paraformaldehyde. Representative fluorescent images were taken at a FLoid Cell Imaging Station.

### 2.12. Statistical Analysis

All the data presented represent the results of at least three independent experiments, and all data are presented as dots or mean or median ± SD or ± SEM. Statistical analysis was performed using two-tailed, unpaired Student’s *t*-test and ANOVA with post-hoc Tukey HSD test. The equal variance of the sample groups was tested by an F-test. * denotes *p* < 0.05, ** denotes *p* < 0.01.

## 3. Results and Discussion

### 3.1. Loss of RetSat Does Not Alter the Regeneration Program in the Tibialis Anterior Muscle Following Cardiotoxin Injury

**Figure 1:**
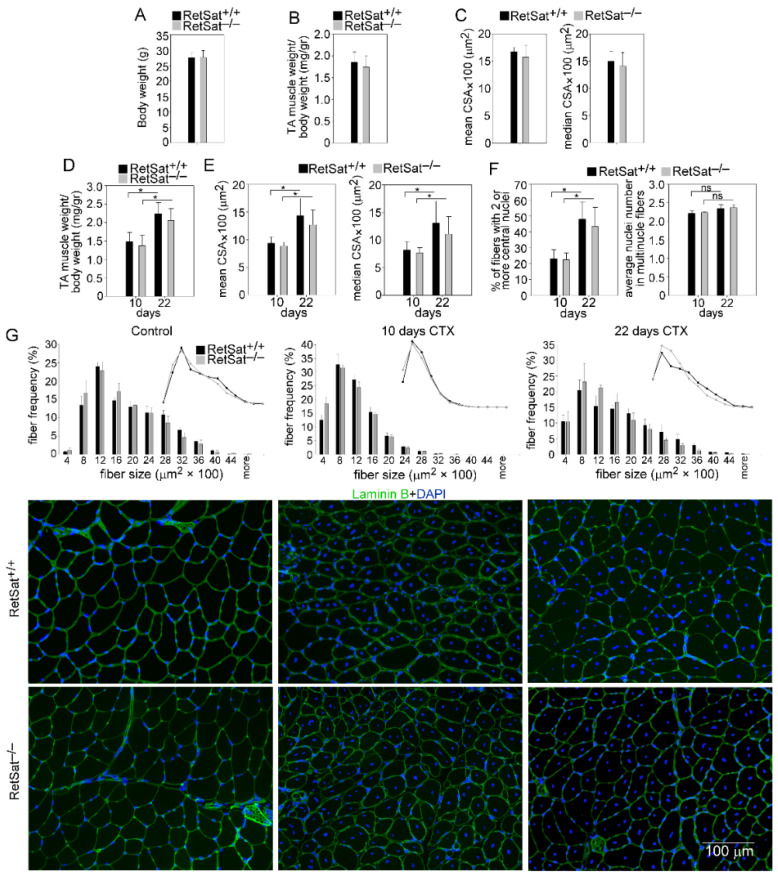
Normal muscle growth and regeneration in RetSat-deficient mice. (**A**) Body weight and (**B**) muscle weight/body weight ratio in RetSat^+/+^ and RetSat^−/−^ untreated mice. (**C**) Mean and median myofiber cross-sectional area (CSA) of the TA muscles in RetSat^+/+^ and RetSat^−/−^ untreated mice. (**D**) Muscle weight/body weight ratio of the regenerating TA muscles at days 10 and 22 post-CTX-injury in RetSat^+/+^ and RetSat^−/−^ mice. (**E**) Mean and median myofiber CSAs of the regenerating TA muscles at days 10 and 22 post-CTX-injury in RetSat^+/+^ and RetSat^−/−^ mice. (**F**) Percentage of newly formed myofibers containing two or more central nuclei, and mean number of central nuclei per fiber at days 10 and 22 post-CTX-injury in RetSat^+/+^ and RetSat^−/−^ mice. (**G**) Myofiber size distribution in the control and regenerating RetSat^+/+^ and RetSat^−/−^ TA muscles with their representative immunofluorescence pictures of laminin (green) and DAPI (blue) nuclear staining. ImageJ software was used to examine 500 or more myofibers in each sample. Scale bars, 100 µm. The data are presented as a mean ± SEM (*n* = 6). Asterisks indicate statistical significance (* *p* < 0.05); ns, not significant.

To study the possible role of RetSat in muscle development, we compared the muscle weights and the myofiber CSAs of control and CTX-treated TA muscles from RetSat^+/+^ and RetSat^−/−^ mice. There was no significant difference between the body and TA muscle weights of RetSat^+/+^ and RetSat^−/−^ mice (Figure 1A,B). The mean and median CSA of the control TA muscles were also similar between the two strains (Figure 1C), indicating that embryonic TA muscle development is normal in the RetSat-deficient mice.

To investigate the effect of RetSat ablation on skeletal muscle regeneration, we induced muscle damage by injecting CTX into the TA muscles and measured their weights and the mean and median myofiber CSAs at days 10 and 22 post-injury. The weights and CSAs of the regenerating muscles were also similar between the two strains (Figure 1D,E); neither did we find a difference in the number of newly formed fibers with 2 or more central nuclei or in the mean number of central nuclei per fiber, which are indicators of myoblast fusion during the muscle regeneration, between RetSat^+/+^ and RetSat ^–/–^ mice determined at days 10 and 22 post-injury (Figure 1F). The fiber size distribution of the muscles before and after injury was also similar between RetSat^+/+^ and ^–/–^ mice (Figure 1G).

**Figure 2:**
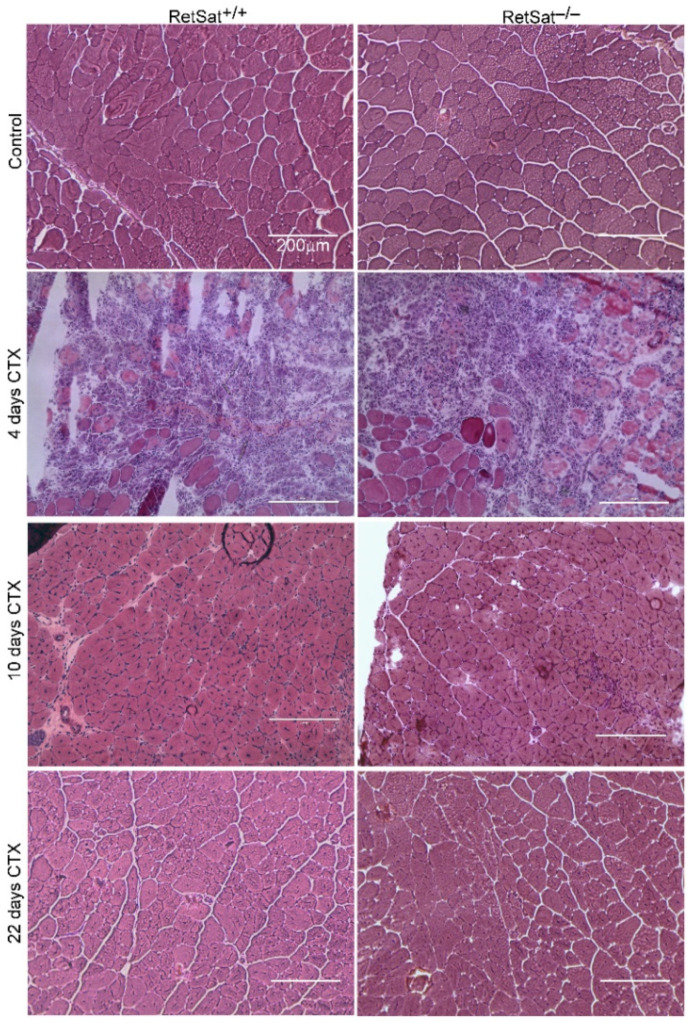
Time-dependent histological morphology of the TA muscles in RetSat^+/+^ and RetSat^−/−^ mice following CTX-induced injury. Injection of 50 μL of 12 μM CTX into the TA muscles was used to trigger muscle damage. Representative H&E-stained cross-sections of the TA muscles from RetSat^+/+^ and RetSat^−/−^ mice before and 4, 10, and 22 days after CTX treatment (*n* = 4). Scale bars, 200 μm.

Histological examination revealed no obvious morphological difference between the control muscles of RetSat^+/+^ and RetSat^−/−^ mice. On day 4, the regenerating muscles of both the wild-type and RetSat^−/−^ mice displayed local necrosis and abundant leukocyte infiltration (Figure 2). By day 10, most of the necrotic tissue was cleared from the muscles, and by day 22 post-injury, the gross histological architecture of the muscles of both RetSat^+/+^ and RetSat^−/−^ mice had been largely restored, and necrotic fibers were no longer visible.

**Figure 3:**
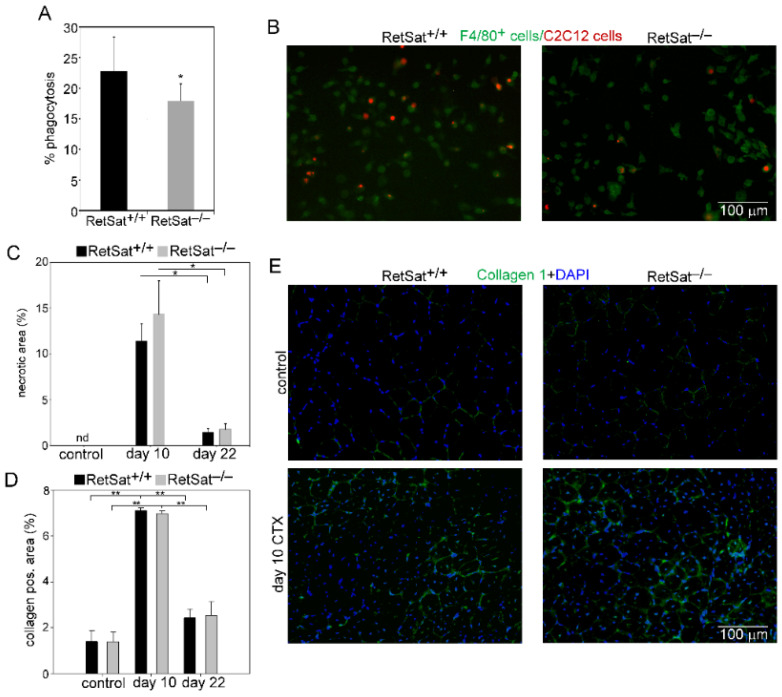
Unaltered necrotic cell removal and collagen deposition in the TA muscles of RetSat-null mice following CTX-induced damage. Decreased in vitro phagocytosis of necrotic C2C12 myoblasts by muscle-derived RetSat^−/−^ F4/80^+^ cells determined (**A**) by FACS analysis and (**B**) by visualizing the number of engulfed cells. (**C**) Necrotic regions in the control and regenerating muscles of RetSat^+/+^ and RetSat^−/−^ mice at days 0, 10, and 22 post-CTX injury. (**D**) Quantification of collagen 1-positive regions in control and 10- and 22-day regenerating muscles of RetSat^+/+^ and RetSat^−/−^ mice. (**E**) Representative immunofluorescence pictures of type 1 collagen (green) and DAPI (blue) nuclear staining in control and 10-day regenerating TA muscles of RetSat^+/+^ and RetSat^−/−^ mice. Scale bars, 100 µm. All data are expressed as mean ± SD (*n* = 4). Asterisks indicate statistical significance (* *p* < 0.05, ** *p* < 0.01); nd, not detected.

Previously, we detected lower phagocytic capacity of the Mϕs in RetSat^−/−^ mice [25] and observed a similar finding when we used necrotic myoblasts as target cells in the in vitro phagocytosis assay performed by muscle-derived Mϕs (Figure 3A,B); therefore, we determined the sizes of the necrotic areas in control and regenerating TA muscles. As shown in Figure 3C, we detected similar necrotic area sizes between the two strains of mice, indicating that the in vivo clearance of dead fibers is not affected by the loss of RetSat.

During muscle repair, the deposition of extracellular matrix proteins is transiently increased, which is required for the regulation of SC, and for myoblast proliferation and differentiation [31]. Therefore, we decided to determine the amount of collagen 1 in the control and regenerating TA muscles. In both mouse strains, there was a temporal increase in collagen 1 deposition at day 10 post-injury, which decreased by day 22, as compared to their own non-regenerating muscles; however, there was no significant difference between the two strains (Figure 3D,E).

**Figure 4:**
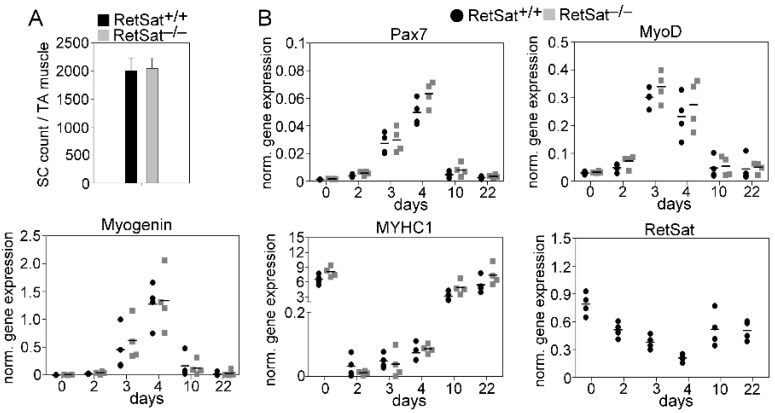
(**A**) The number of SC cells in the TA muscles at day 4 following CTX-induced injury, and (**B**) mRNA expression of RetSat and of various myogenic genes in the control and regenerating TA muscles of RetSat^+/+^ and RetSat^−/−^ mice determined by qRT-PCR (*n* = 4).

To assess the possible impact of RetSat ablation on gene expression and SC cell proliferation and differentiation in the control and regenerating TA muscles, we determined the number of SC cells (Figure 4A) and the expression of myogenic genes, such as the Pax7, MyoD, and myogenin transcription factors involved in myoblast proliferation and differentiation and that of the myosin heavy chain (MYHC)1 differentiation marker. During muscle regeneration, the mRNA expression of Pax7, MyoD, and myogenin transiently increased, while that of RetSat and MYHC1 transiently decreased in the TA muscles; however, with the exception of RetSat, there was no significant difference in their expression between the two mouse strains (Figure 4B).

Altogether, these data imply that loss of RetSat does not affect the number of SCs in the skeletal muscle, nor does the loss of RetSat impact skeletal muscle development or regeneration.

### 3.2. Decreased Recruitment of Mϕs and Neutrophils after Injury in the Absence of RetSat

**Figure 5:**
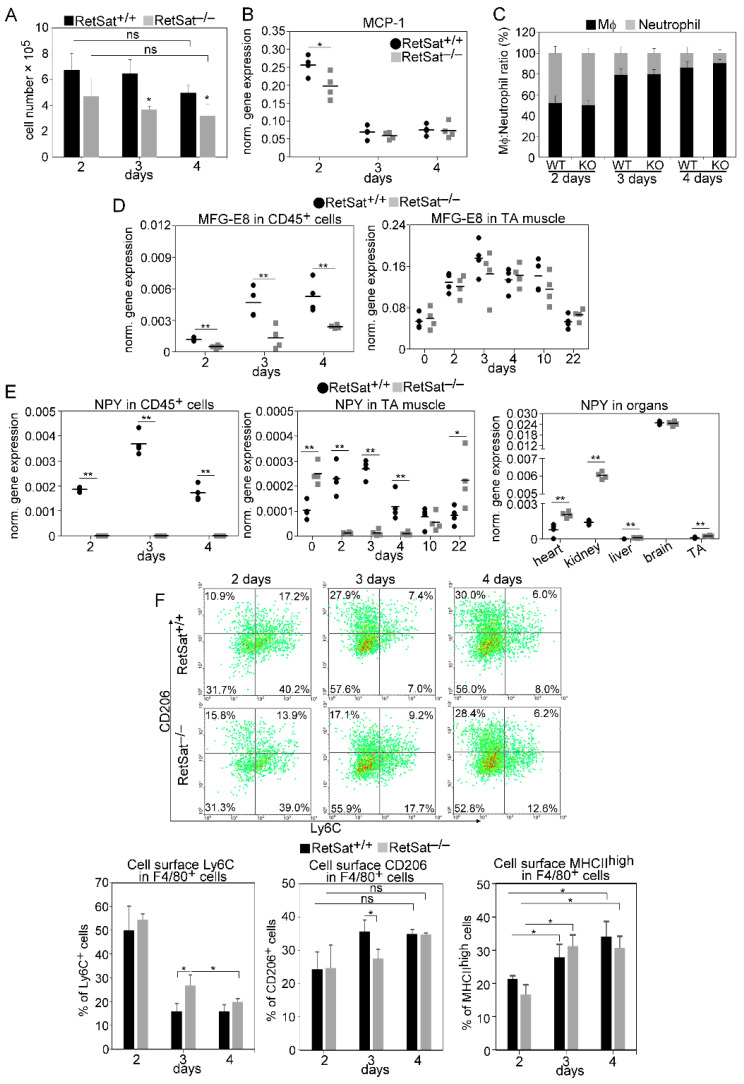
Decreased tissue infiltration and altered polarization of muscle-derived F4/80^+^ cells in the absence of RetSat. (**A**) The number of CD45^+^ leukocytes per injured muscle and (**B**) MCP-1 mRNA expression levels of muscle-derived CD45^+^ leukocytes from wild-type or RetSat-null mice, determined by qRT-PCR following CTX-induced injury (*n* = 4). (**C**) Ratio of anti-F4/80 antibody-stained Mϕs and anti-Ly6G/Ly6C (GR-1)-stained neutrophils within the CD45^+^ leukocyte populations in the TA muscles from RetSat^+/+^ and RetSat^−/−^ mice during the first 4 days of regeneration following CTX-induced injury (*n* = 3). (**D**) Expression of MFG-E8 mRNA and (**E**) of NPY mRNA in muscle-derived CD45^+^ leukocytes, in total TA muscles, and various organs of RetSat^+/+^ and RetSat^−/−^ mice, determined by qRT-PCR following CTX-induced injury (*n* = 4). (**F**) Representative scatter plots of CD206- and Ly6C-stained muscle-derived F4/80^+^ cells and the percentages of Ly6C^+^, CD206^+^, and MHCII^high^ cells within the muscle-derived F4/80^+^ population determined at the indicated days following CTX-induced injury in the TA muscles of RetSat^+/+^ and RetSat^−/−^ mice (*n* = 3). Asterisks indicate statistical significance (* *p* < 0.05, ** *p* < 0.01); ns, not significant.

Following injury, muscle repair is initiated by the migration of inflammatory cells to the injury site. To determine the composition of leukocytes in the early phase of muscle regeneration, we performed a flow cytometric analysis of magnetically separated CD45^+^ cells from collagenase digested muscles. In accordance with previous observations, we observed early infiltration of neutrophils at day 2 post-injury, followed by an increasing number of Mϕs at days 3 and 4 in wild-type mice. However, in the absence of RetSat, a significantly decreased number of CD45^+^ cells infiltrated the injured muscle (Figure 5A). In line with this observation, at day 2 post-injury, a significantly decreased gene expression level of monocyte chemoattractant protein-1 (MCP-1) was detected (Figure 5B), whereas the neutrophil/Mϕ ratios did not change (Figure 5C).

### 3.3. Myoblasts Compensate for Attenuated MFG-E8 Levels of Macrophages in RetSat-Null Mice

To investigate the impact of RetSat ablation on the polarization of Mϕs and on their gene expressions, CD45^+^ cells from collagenase-digested regenerating muscles were magnetically separated at days 2, 3, and 4 post-injury and stained for the cell surface marker proteins F4/80, Ly6C, CD206, and MHCII. In addition, the expression of their genes was determined by quantitative PCR. Since to our surprise, based on the relative disappearance of necrotic areas (Figure 3C), loss of RetSat in vivo did not seem to affect efferocytosis during skeletal muscle regeneration, we first checked whether muscle-derived CD45^+^ cells from the RetSat-null mice altered MFG-E8 levels. As seen in Figure 5D, expression of MFG-E8 mRNA within the CD45^+^ cells gradually increased until day 4 following cardiotoxin-induced injury in both mouse strains. In accordance with our previous observations [25], the muscle-derived CD45^+^ cells in the RetSat-null mice also expressed significantly less MFG-E8. This finding is in accordance with the results of the in vitro phagocytosis assay, which demonstrated a decreased long-term efferocytosis capacity for the muscle-derived RetSat null macrophages (Figure 3A,B).

However, in the regenerating muscle, we found no alterations in the levels of MFG-E8 mRNA in the RetSat-null mice as compared to their wild-type littermates (Figure 5D), indicating that very likely myoblasts (the only cell type besides Mϕs that is known to produce MFG-E8 in regenerating skeletal muscle) fully compensate for the attenuated MFG-E8 production of Mϕs in the RetSat-null mice. In fact, using the data derived from the NPY mRNA expression in the CD45^+^ cells and in the total muscle (Figure 5E), we could estimate that in the wild-type regenerating muscles, less than 1% of the MFG-E8 is derived from CD45^+^ cells. Since MFG-E8 is secreted into the tissue environment, myoblast-derived MFG-E8 is expected to become available for phagocytosing cells as well. Thus, our data indicate that in the regenerating muscles, independent of RetSat expression, macrophage-derived MFG-E8 has no limiting effect on the phagocytic capacity of macrophages. This observation partly explains why the in vivo efferocytosis did not change in the regenerating muscles of RetSat-null mice.

### 3.4. Altered NPY Levels Both in Mϕs and in the Skeletal Muscle of RetSat-Null Mice

Next, we assessed the levels of expression of NPY in muscle-derived CD45^+^ cells. NPY has anti-inflammatory functions [26,27], and was also shown to promote angiogenesis [32]. NPY mRNA levels in CD45^+^ cells from the muscles of wild-type mice increased until day 3 following CTX-induced injury, and then they started to decrease. However, in accordance with our previous findings [25], muscle-derived CD45^+^ cells from RetSat-null mice lacked significant expression of NPY (Figure 5E).

Skeletal muscle does not express NPY mRNA or protein [33], but sympathetic neurons do, and they co-release the NPY with noradrenaline following stimulation [34]. Accordingly, we detected expression of NPY mRNA in control muscles, and in muscles from day 10 following CTX-induced injury in both wild-type and RetSat-null mice (Figure 5D). In this context, it is worth noting that sympathetic neurons regulate immune functions via NPY [35] and were reported to facilitate muscle repair [36]; we detected a significantly enhanced amount of NPY mRNA in the muscles of control and day-22 post-injury RetSat-null mice. The finding was similar when we checked the NPY levels in other organs, except for the brain, where NPY mRNA expression was similar. These data indicate that the loss of RetSat affects the mRNA expression of NPY not only in Mϕs but also in the sympathetic neurons innervating various tissues.

In addition, in the muscles of wild-type mice, expression of NPY mRNA increased until day 3 following injury, then gradually decreased, whereas no expression of NPY mRNA was detected during this time period in the muscles of RetSat-null mice. Since the CD45^+^ cells of RetSat-null mice do not express NPY, this transient increase in the wild-type muscle could be a result of infiltrating NPY-expressing CD45^+^ cells.

### 3.5. A Transient Delay in the M1/M2 Phenotypic Switch in Mϕs of RetSat-Null Mice during the Muscle Regeneration Process

Since proper timing of the M1/M2 phenotypic change of Mϕs is central in guiding muscle regeneration, we followed the process by determining the time-dependent changes in the expression of M1- and M2-specific cell surface markers of Mϕs, and in the expression of genes of the CD45^+^ cells. Though the loss of RetSat did not affect the in vivo efferocytotic capacity of Mϕs, known to result in an altered polarization of Mϕs, at post-injury day 3, we detected a delayed generation of Ly6C^−^ CD206^+^ Mϕs from the Ly6C^+^ RetSat^−/−^ NPY null pro-inflammatory cells (Figure 5F). However, this delay disappeared by day 4, perhaps due to the high myoblast-derived MFG-E8 level that also promotes the M1/M2 conversion, and the appearance of CD206^+^ macrophages [37]. At the same time, the formation of the MHCII^high^ expressing cells was not affected.

### 3.6. mRNA Expression of RetSat, Various Cytokines, and Growth Factors in the CD45^+^ Macrophages Derived from the Regenerating TA Muscles of RetSat^+/+^ and RetSat^−/−^ Mice

**Figure 6:**
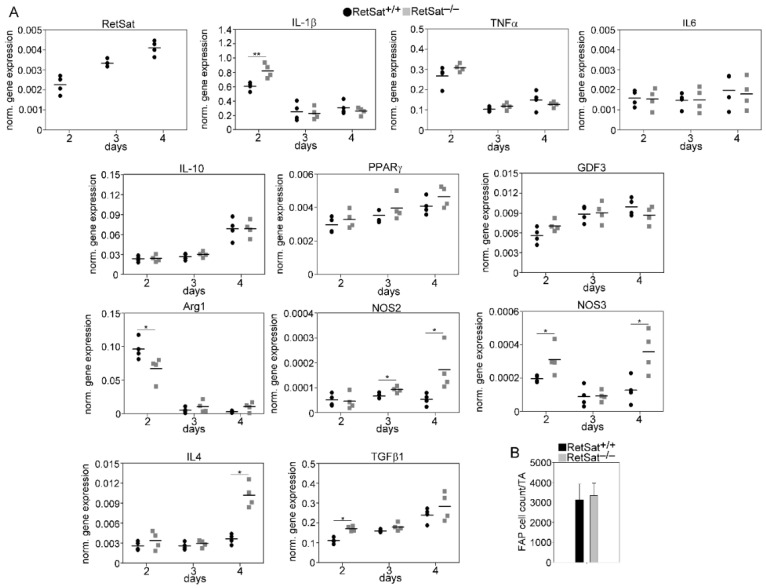
(**A**) mRNA expression of RetSat and of various M1-and M2-marker genes in CD45^+^ cells isolated from TA muscles determined by qRT-PCR at days 2, 3, and 4 post-injury (*n* = 4). Asterisks indicate statistical significance (* *p* < 0.05, ** *p* < 0.01). (**B**) The number of FAP cells in CTX-injected TA muscles determined at day 4 following injury. Data are expressed as mean ± SD.

In accordance with our previous publication [38], the expression of RetSat mRNA increased in the engulfing CD45^+^ cells (Figure 6A). In line with the lack of immunosuppressive NPY production, at day 2 post-injury as compared to their wild-type counterparts, CD45^+^ cells from RetSat-null mice expressed an increased amount of pro-inflammatory IL-1β, a cytokine that strongly contributes to SC cell proliferation and differentiation [39]. However, we did not find alterations in the expression of other pro-inflammatory cytokines, such as TNF-α or IL-6, nor did we find a change in the appearance of the mRNA expression of the M2-like IL-10, PPARγ, or GDF3 genes. However, the mRNA expression of three enzymes known to determine NO release, arginase 1 (Arg1), nitric oxide synthase 3 (NOS3), and the inducible iNOS, did change in such a way that they promoted long-term NO production. Proper GDF3 production might be associated with normal myoblast differentiation in RetSat-null mice. Prolonged NO production, together with elevated IL-1µ levels, on the other hand, might promote SC cell activation in the presence of fewer Mϕs [40], might contribute to the initiation of angiogenesis in the absence of NPY [41], and might enhance efferocytosis by promoting the appearance of PS on the surface of apoptotic cells [42].

Previous studies have demonstrated that, in addition to Mϕs, eosinophils are also essential for proper muscle repair by producing IL-4 during muscle regeneration [43]. IL-4 contributes to the proper M2-like polarization of Mϕs [44]; but most importantly, it triggers the proliferation of FAP cells [43]. FAP cells work side by side with Mϕs to properly clear the necrotic cells and contribute to myogenesis [43,45]. In addition to IL-4, macrophage-derived TGF-β1 is also involved in the generation of FAP cells, and the number of FAPs correlates with the TGF-β1 levels [46]. In this context, it is worth noting that we observed significantly elevated TGF-β1 and IL-4 mRNA expression levels in the RetSat-null CD45^+^ cells at post-injury days 2 and 4, respectively, and as a result, we detected unaltered FAP cell numbers in the RetSat null muscle during regeneration (Figure 6B).

## 4. Conclusions

Previous investigations have shown that strong intercellular crosstalk between various populations of cells in the regenerating muscle drives and balances the process of skeletal muscle repair [47,48]. Our data presented in this paper demonstrate that through this crosstalk, several compensatory mechanisms are induced in the regenerating muscles of RetSat-null mice to replace the impaired functions of the Mϕs, which is attributed to their attenuated MFG-E8 production, and to their lack of NPY expression. Thus, high levels of MFG-E8 produced by the regenerating muscles generally obviate the need for MFG-E8 release by macrophages for proper efferocytosis, independently of the loss of RetSat. Higher levels of IL-1β and NO produced by CD45^+^ cells replace the need for NPY (which might appear at the site of repair from the sympathetic neurons as well) and promote sufficient SC cell proliferation, despite the presence of fewer neutrophils and macrophages at the regeneration sites of the RetSa-null muscles. Increased production of TGF-β and IL-4 by CD45^+^ leukocytes, on the other hand, maintains the proper FAP cell proliferation, contributing to the proper in vivo dead cell clearance. Our data cannot distinguish whether these alterations are the result of RetSat ablation or if they would also be induced under other circumstances when fewer neutrophils and macrophages infiltrate the damaged skeletal muscle area. Nevertheless, as a result of the adapted crosstalk, normal skeletal muscle repair was observed following CTX-induced injury in the RetSat-null mice.

## Data Availability

The data presented in this study are available upon request from the corresponding author.

## Supplementary Materials

The following supporting information can be downloaded at: https://www.mdpi.com/article/10.3390/cells11081333/s1, Figure S1: The expression of neomycin resistance expression cassette (neoR), replacing exon 1 of RetSat gene in various tissues isolated from wild-type and full-body RetSat−/− mice determined by RT-qPCR.
