# Supplementary material for: Regenerating Skeletal Muscle Compensates for the Impaired Macrophage Functions Leading to Normal Muscle Repair in Retinol Saturase Null Mice
Source: Cells. 2022 Apr 13;11(8):1333. doi: 10.3390/cells11081333 (PMC9028072; doi:10.3390/cells11081333)
Supplement: Supplementary file 1 [file cells-11-01333-s001.zip › cells-1600311-supplementary.pdf]

Supplementary Materials

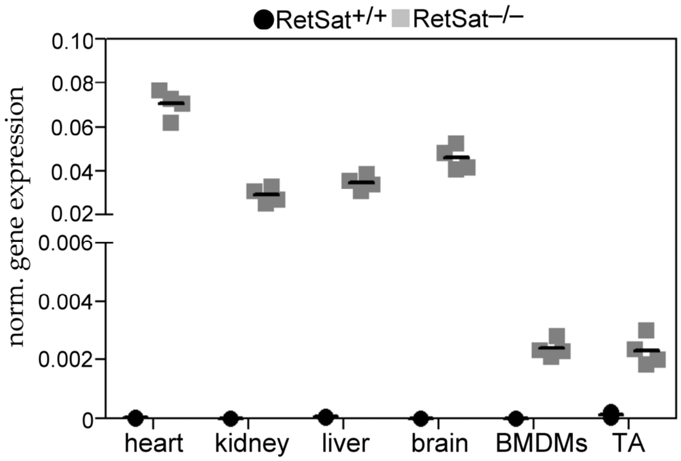

Figure S1. The expression of neomycin resistance expression cassette (neoR), replacing exon 1 of RetSat gene in various tissues isolated from wild-type and full-body RetSat–/– mice determined by RT-qPCR.

The homozygous RetSat$^{-/-}$ mice were generated by homologous recombination in embryonic stem cells toreplace exon 1 of the wild-type RetSat gene with a neoR cassette. The neoR cassette replaced an approximate 2 kbp of the RetSat gene, including exon 1 containing the translation start ATG. The mutated gene is expressed and neoR can be detected by RT-qPCR but there is no functional RetSat protein expression [29]. n=4.
